# A 10-year survival rate of tapered self-tapping bone-level implants from medically compromised Korean patients at a maxillofacial surgical unit

**DOI:** 10.1186/s40902-023-00401-w

**Published:** 2023-10-06

**Authors:** Buyanbileg Sodnom-Ish, Mi Young Eo, Myung Joo Kim, Soung Min Kim

**Affiliations:** 1 Department of Oral and Maxillofacial Surgery, Dental Research Institute, School of Dentistry, Seoul National University, Seoul, Korea; 2 Department of Prosthodontics, Dental Research Institute, School of Dentistry, Seoul National University, Seoul, Korea

**Keywords:** Dental implants, Maxillofacial surgical unit, Medically compromised patient, Survival rates, Tapered bone-level implant

## Abstract

**Background:**

The 10-year survival rate of dental implants in healthy subjects is 90–95%. While in healthy individuals, dental implants have become commonplace to solve problems of edentulism, whether dental implant treatment is optimal in patients with systemic disease remains unclear. The purpose of this study is to investigate the clinical outcomes of tapered, sand-blasted, and acid-etched internal submerged dental implants installed in medically compromised patients in our maxillofacial surgical unit.

**Methods:**

A total of 1019 Luna® dental implants were placed in 333 patients at the Department of Oral and Maxillofacial Surgery, Seoul National University Dental Hospital. Kaplan–Meier survival estimates after 10 years of follow-up were computed for healthy vs. medically compromised patients.

**Results:**

The 10-year follow-up survival rate of 1019 Luna® dental implants in the Korean maxillofacial surgical unit was 97.0% with a mean follow-up of 41.13 ± 35.13 months (0–120 months). The survival rate was 97.0%, in which 31 implants were failed during the follow-up. Cumulative 10-year implant survival rates were 99.4% in healthy individuals without systemic disease and 95.9% in patients with systemic disease.

**Conclusions:**

Comparable success and survival rates were achieved with those of implants in healthy patients. Preoperative general health assessments including laboratory test results and checking the previous medication records are essential in diagnosing any unrecognized conditions for improved implant success rates in medically compromised patients.

## Background

The long-term survival rate of dental implants in healthy individuals is reported to be around 90–95% over a 10-year period [1, 2]. While dental implants have become a common treatment for edentulism in healthy individuals, there is still ongoing debate regarding their preference in medically compromised patients. To ensure optimal candidacy for elective surgical procedures like dental implant installation, it is recommended to restrict eligibility to patients classified as ASA (American Society of Anesthesiologists) grade I or grade II [3]. Emphasizing systemic disease control is crucial, and establishing an individualized medical equilibrium prior to implant therapy is essential. The potential benefits provided by dental implants should outweigh the patient’s surgical risks.

In the existing literature, only a limited number of absolute contraindications to dental implants have been identified, although certain conditions may increase the risk of implant failure and complications. Absolute contraindications for implant therapy include recent heart attack, stroke, cardiac transplant or valvular prosthesis surgery, severe bleeding tendency, profound immunosuppression, ongoing cancer treatment, substance abuse, psychiatric disorders, and the use of intravenous bisphosphonates [4]. However, there is currently insufficient evidence either supporting or refuting these presumed contraindications. Controversy surrounds the potential risk of complications and implant failure associated with dental implant installation in medically compromised patients [5].

The purpose of this study was to investigate the clinical outcomes of tapered, sand-blasted, and acid-etched internal submerged bone-level dental implants (Luna®, Shinhung Co., Seoul, Korea) installed in medically compromised patients in the maxillofacial surgical unit.

## Methods

The patients were treated from August 2011 to July 2021 in the Department of Oral and Maxillofacial Surgery, Seoul National University Dental Hospital (SNUDH), for a period of 10 years. The study included 333 patients with 1019 dental implants. The study protocol and access to patient records were approved by the Institutional Review Board of Seoul National University (IRB No. S-D20200007), Seoul, Korea. All implant placement surgical procedures were carried out by one surgeon at SNUDH. The minimal follow-up period was at least 3 months after prosthesis delivery.

### Inclusion criteria

- Patients who underwent dental implant (Luna®, Shinhung Co., Seoul, Korea) installation in the maxillofacial unit of SNUDH.
- Patients with controlled systemic disease who received dental implant installation.
- Patients with complete medical data, including the clinical and radiographic findings.

### Exclusion criteria

- Patients with uncontrolled systemic disease.
- Previous history of trauma in the oral and maxillofacial area.
- Incomplete data.
- Patients who were lost during follow-up.

### Treatment procedures

The treatment involved the utilization of the submerged procedure. Under local anesthesia, a single oral and maxillofacial surgeon performed implant installation following the Luna® implant surgical protocol. All implants demonstrated good primary stability. The re-entry procedure was performed approximately 3 to 6 months after the initial implant installation. Once the soft tissue had adequately healed, the prosthesis was fabricated after 2 to 4 months.

### Implant data

We carried out vertical and horizontal bone augmenting procedures such as guided bone regeneration, block bone grafting, sinus lifting, and socket lifting based on each case’s remaining bone quality and the type of bone graft material. We installed the implants based on the third ITI Consensus Conference, based on the period between tooth extraction and implant placement [6].

### Implant success criteria

Implant success criteria were based on the ICOI, Pisa, Consensus Conference 2007, which included the absence of pain and tenderness on function, absence of mobility, less than a 2-mm radiographic bone loss from initial surgery, and no exudates history [7].

### Implant failure criteria

The criteria are any of the following: radiographic bone loss exceeding half the length of the implant, pain on function, mobility, uncontrolled exudate, and no longer in mouth.

### Implant survival criteria

Evaluation during the follow-up period for each patient included the clinical and radiographic situations such as implant stability, bone loss around the implants, signs of infection, and the level of bone around the implants.

### Study variables

Various clinical events were recorded, including the implant placement date, loading time, last follow-up period, and implant failure or removal date. The primary outcome variable for this study was implant failure. Survival time referred to the duration from implant installation to either implant removal or the last follow-up for surviving implants. The study variables were divided into two groups: healthy individuals without systemic disease and patients with medical conditions. Detailed information about the variables investigated can be found in Tables 1, 2, and 3. In our study, we did not include any uncontrolled variable such as previous history of trauma in the oral and maxillofacial area. Among 333 patients, only one patient had a history of car accident which did not affect the oral and maxillofacial area. Regardless of his trauma history, the patient had concomitant systemic conditions including hypertension and bronchial asthma, which met the inclusion criteria. In addition, we found successful treatment outcomes in this patient. Therefore, we believe that the previous trauma history in this case will not cause any bias in the result.

**Table 1:** Implant length distribution

Implant length	Number	%
Maxilla		
7	56	5.45
8.5	239	23.45
10	209	20.51
11.5	1	0.10
Mandible		
7	82	8.05
8.5	266	26.10
10	142	13.93
11.5	3	0.30
Facial prosthesis		
7	5	0.50
8.5	13	1.27
10	0	0
11.5	3	0.29
Total	1019

**Table 2:** Implant diameter distribution

Implant diameter	Number	%
Maxilla		
3.5	128	12.56
4	335	32.87
4.5	41	4.02
5	1	0.10
Mandible		
3.5	66	6.48
4	392	38.47
4.5	29	2.85
5	6	0.59
Facial prosthesis		
3.5	6	0.59
4	15	1.47
4.5	0	0
5	0	0
Total	1019

**Table 3:** Patient demographics

Variables	Number	%
Male	134	40.24
Female	199	59.76
Past medical history (PMH)		
Cardiovascular disease (CVD)	87	26.13
Diabetes mellitus (DM)	18	5.40
CVD & DM	31	9.30
Osteoporosis	34	10.21
Hyperlipidemia	21	6.31
Oral tumors	18	5.41
Other cancers	15	4.50
Sinusitis and rhinitis	15	4.50
Autoimmune disorder	10	3.00
Hepatitis B virus	5	1.50
Mental disorder	5	1.50
Dementia	4	1.20
Endocrine diseases	4	1.20
Kidney disease	3	0.90
Hepatitis C virus	2	0.60
Lung disease	2	0.60
Liver disease	2	0.60
Macular degeneration	1	0.30
Knee cartilage surgery	1	0.30
Insomnia	1	0.30
Thrombocytopenia	1	0.30
Prostate hypertrophy,	1	0.30
Fallopian tube extraction	1	0.30
Gastric ulcer	1	0.30
Midfacial deformity	1	0.30

**Fig. 1:**
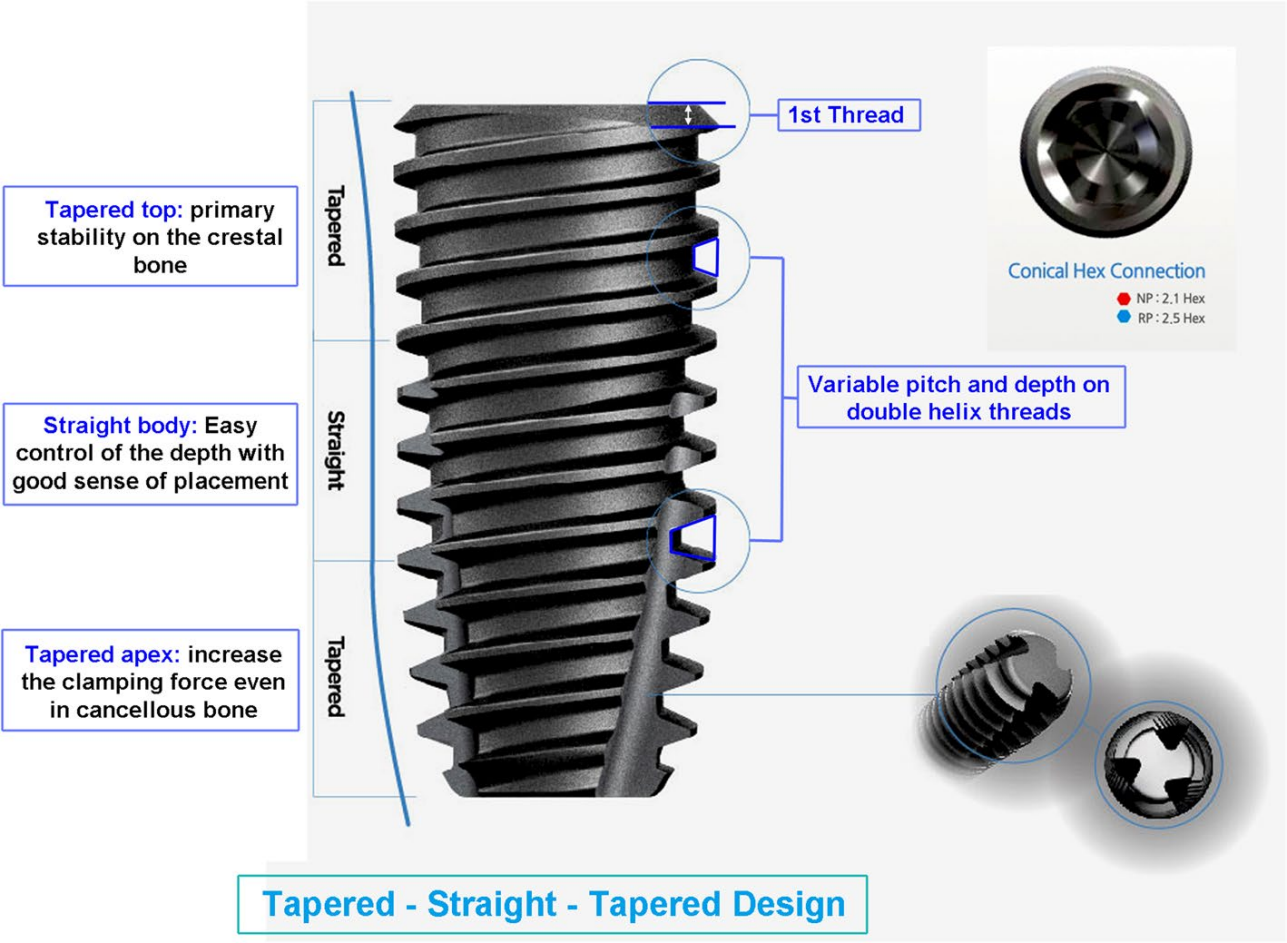
Macroscopic design of the Luna® implant system used in this study

**Fig. 2:**
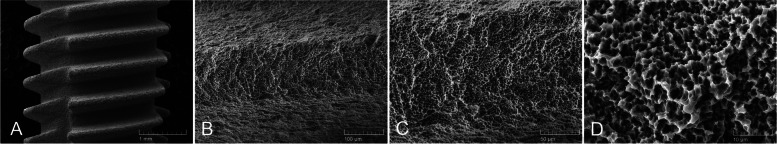
Scanning electron microscopy (SEM) view of the surface morphology of the self-tapped bone level, sand-blasted, and acid-etched surface. The SEM was operated at 20 kV. The secondary electron (SE) detection mode was used for the ultrastructural surface analysis. SEM × 60 magnification view (**a**), × 500 magnification view (**b**), × 1000 magnification view (**c**), and × 5000 magnification view (**d**)

The primary type of implant used in this study was the Luna® (Shinhung Co., Seoul, Korea) self-tapped bone-level implant, depicted in Fig. 1. The Luna® dental implants have a sand-blasted and acid-etched surface with a roughness of 2.5 μm or higher, resulting in a 20% improvement in bone healing period and cell response (Fig. 2). The optimal number of implants for each patient was determined based on the prosthesis design and the extent of edentulism.

### Statistical analysis

Statistical analysis was performed using SPSS (version 23 IBM®, NY, USA), and implant-related data were calculated. The Kaplan–Meier analysis was used for the description of survival rates.

## Results

This study enrolled 333 patients with 1019 implants. The mean age was 64.66 years old (male 65.38; female 64.16) at the time of the surgery. Out of 1019 implants, 505 implants were installed in the maxilla, 493 implants in the mandible, and 21 implants in the infraorbital rim and zygoma for facial prosthesis. 83% of the cases used augmentation, while in 17% no augmentation was carried out. Of the 333 patients, 110 (33.03%) did not have systemic disease, while 223 (69.97%) were medically compromised.

**Fig. 3:**
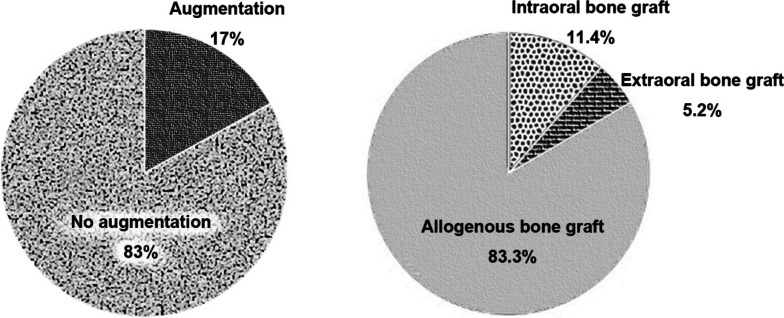
Bone augmentation data. Bony augmentation procedures were applied in 17% of the implants. Among the bone-augmented cases, allogenous bone material was further preferred with a rate of 83.3%, followed by intraoral bone grafting and finally extraoral bone grafting

According to the third ITI Consensus, the majority of the implant’s installations were type IV (70.4%), which were placed more than 6 months after tooth extraction. The remaining implants were type I (7.2%), type II (3.1%), and type III implant placement with partial bone healing (19.3%). Bone grafting procedures were applied in 17% of the implants in this study. Among the bone-grafted cases, and allogenous bone material was mostly used (83.3%), followed by intraoral bone grafting and finally extraoral bone grafting (Fig. 3).

Of 31 failed implants at a 10-year follow-up, 6 failed implants were in patients with cardiovascular disease (CVD); 4 implants in patients with CVD and diabetes mellitus (DM); 3 implants in patients with squamous cell carcinoma, other tumors, and odontogenic keratocyst; and 1 implant failure each in patients with hyperlipidemia, osteoporosis, thrombocytopenia, and mental illness.

**Fig. 4:**
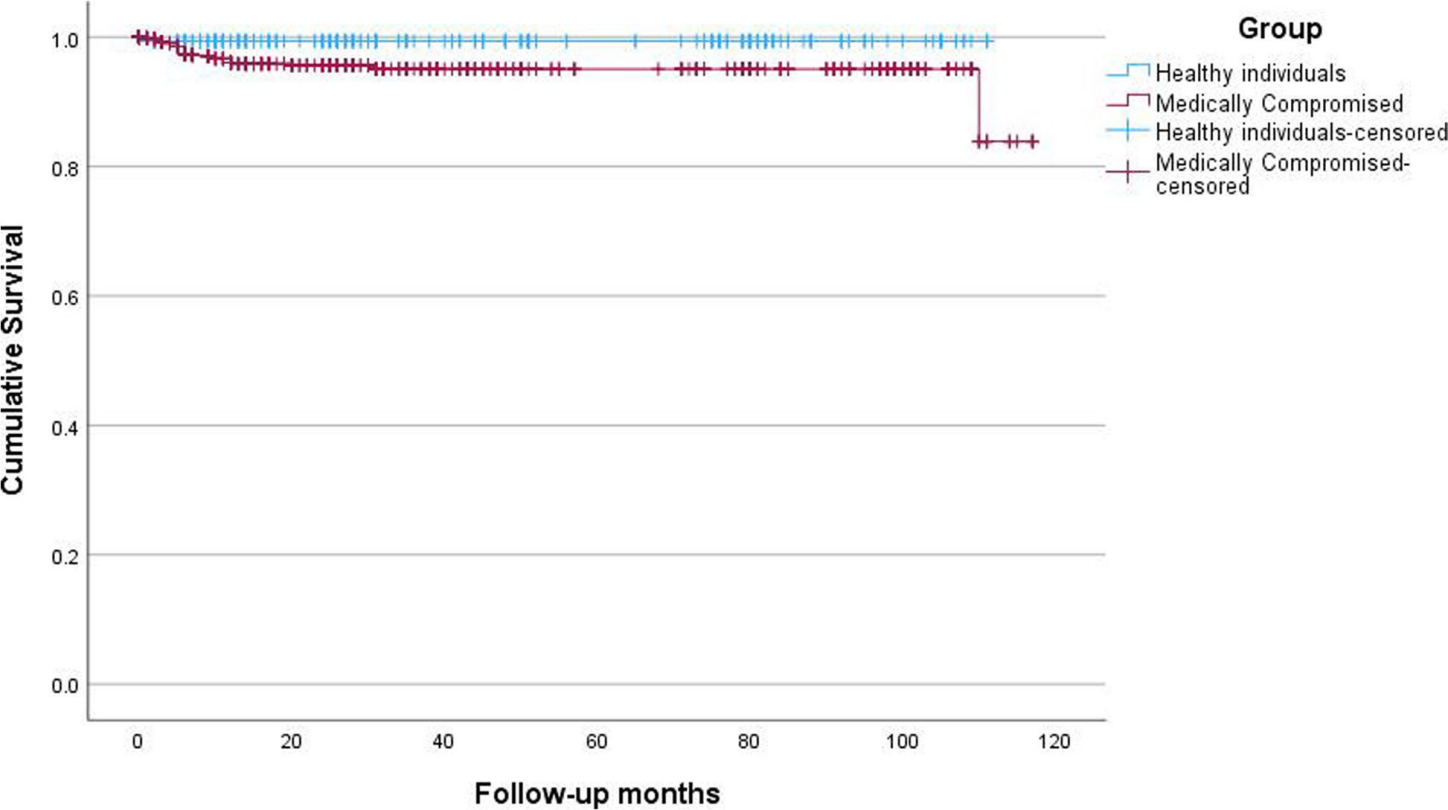
Kaplan–Meier survival curve according to healthy individuals without systemic disease vs. medically compromised patients

Six out of 31 were successfully re-installed with sand-blasted, large grit, and acid-etched surfaced Stella® implants (Shinhung Co., Seoul, Korea). The 10-year follow-up survival rate of 1019 Luna® dental implants in the Korean maxillofacial surgical unit was 97.0% with a mean follow-up of 41.13 ± 35.13 months (range 0 to 120 months). During the follow-up period, there were 31 dental implant failures, leading to an overall survival rate of 97.0%. Over a cumulative period of 10 years, the survival rates of implants were 99.4% for individuals without any underlying systemic disease, and 95.9% for patients with systemic disease (Fig. 4). 7.0-mm dental implants showed a survival of 95.1%, 9.0-mm dental implants showed 96.7%, and 10-mm dental implants showed 98.0%, while 12-mm dental implants showed 100% implant survival at a 10-year follow-up (Table 4). Concerning implant diameter, narrow implants (diameter ≤ 3.5 mm) showed an implant survival rate of 98.5%, 4.0-mm diameter implants showed 96.8%, and 4.5-mm diameter implants showed 94.3%, while 5.0 mm implants showed 100% survival rate. However, only seven 5.0-mm diameter implants were installed, so the validity could be limited (Table 5). There were no statistically significant differences observed in the survival rates of implants based on the diameter (*p* = 0.118) and length (*p* = 0.071) of the implants.

**Table 4:** Implant success and survival rates based on the length

Implant length	Success/failure (total)	Survival rate
7	136/7 (143)	95.1%
8.5	501/17 (518)	96.7%
10	344/7 (351)	98%
11.5	7/0 (7)	100%
Total	988/31 (1019)	97.0%

**Table 5:** Implant success and survival rates based on the diameter

Implant diameter	Success/failure (total)	Survival rate
3.5	197/3 (200)	98.5%
4	718/24 (742)	96.8%
4.5	66/4 (70)	94.3%
5	7/0 (7)	100%
Total	988/31 (1019)	97.0%

## Discussion

The patients presenting for implant treatment at our university dental hospital were geriatric patients, many of whom were medically compromised. The representative cases of successful implant rehabilitation included cases of patients with CVD, lupus arthritis, zygoma implant installation due to total maxillectomy, melanoma, renal and lung cancer, and fibular mandible due to mandibulectomy.

In our study, the cumulative survival rate of 1019 dental implants in medically compromised patients in the maxillofacial surgical unit during a 10-year follow-up period was 97.0%. Our previous study showed a survival rate of 98.1% of 105 sand-blasted and acid-etched surfaced tissue-level dental implants in sixty-one partially and fully edentulous patients at the end of a 5-year follow-up [8].

The importance of quality of life has become increasingly recognized, with oral health playing a vital role in overall well-being. Dental implants have emerged as a preferred option for tooth replacement, particularly for individuals with medical conditions. The prevalence of systemic diseases among elderly patients is commonly observed, which may potentially affect their capacity for bone healing. The demand for dental implant treatments among elderly and medically compromised individuals is steadily rising due to the growing aging population [1]. Studies have indicated that the survival rates and peri-implant health in medically compromised patients are comparable to those of healthy individuals. For instance, a retrospective follow-up study conducted by Millesi et al. found no significant difference in implant survival rates between patients with diabetes (97.3% survival rate) and healthy individuals (98.6% survival rate), as well as between osteoporotic patients (97.3% survival rate) and healthy individuals (97.2% survival rate) [9]. In another study, dental implants in 20 elderly and medically compromised patients resulted in a comparable survival rate of 96.6% in 60 implants [10]. Since the demand for implant rehabilitation is increasing and the current results show a high survival rate of dental implants in medically compromised patients, specific precautions should be taken for each patient and careful follow-up care should be applied in these patients.

**Fig. 5:**
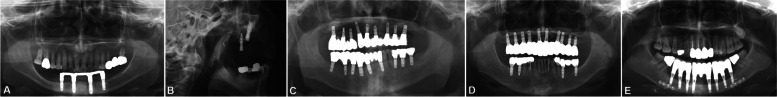
Representative cases of successful S&E and self-tapping implant rehabilitation in a 60-year-old female patient with a history of cardiovascular disease and lupus with #41i, 43i, 31i, 33i implants (**A**); a 74-year-old male patient with orbital implants for silicone facial prosthesis (**B**); a 67-year-old male patient with a history of CVD, DM, renal, and lung cancer with #11i-17i, #21-27i, #41i-47i, and 31i-37i implants (**C**); a 84-year-old female patient with a history of cardiovascular disease, both sinus lifting with guided bone regeneration, onlay bone graft on both side of the mandible with #17i-11i, #21i-27i, #36i-37i, and #46i-47i implants (**D**); a 33-year-old female patient with a history of ameloblastoma, partial mandibulectomy, and reconstruction with radial forearm free flap reconstruction with #42i-45i and #32i-35i implants (**E**)

In our study, we found that the survival rates of implants were 99.4% for individuals without any underlying systemic disease and 95.9% for patients with systemic disease during the cumulative period of 10 years. According to the literature, this comparable rate of implant survival between groups could be explained by the additional procedures, follow-up period, periodontal disease, and ingestion of antihypertensive drugs, which are beneficial for bone formation and remodeling [2]. Another explanation is that all of the patients thoroughly undergo general health assessments, including laboratory test results and checking of the previous medication records in our maxillofacial surgical unit prior to any surgical treatment. The medical conditions that may affect the dental implant treatment may be recognized or not recognized by the patient, in some cases controlled or not controlled by the doctor. This comprehensive evaluation allows us to screen any undiagnosed conditions and promptly refer and consult the patients to the related departments for further investigation and treatment. By closely monitoring their health status and addressing any potential complications, we can ensure timely interventions and optimize their overall well-being. This proactive approach to healthcare significantly contributes overall success and survival of implants (Fig. 5).

Previous study has demonstrated that self-tapping implants exhibit notably greater initial stability when compared to non-self-tapping implants [11]. This can be explained by the high level of the implant to bone contact, facilitated by the compressive threads and the minimal lateral displacement that occurs during implant insertion. As the implant is inserted, the loose trabeculae of bone are compressed, leading to increased density in the areas between adjacent implant threads. This enhancement of local bone characteristics, particularly in regions responsible for primary stability, contributes to a stronger support for the implant. Consequently, the primary stability of the implant is increased.

**Fig. 6:**
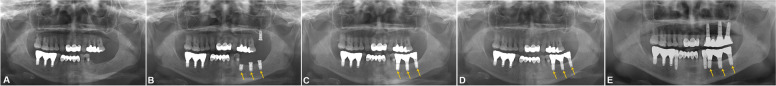
A 10-year follow-up of self-tapping implants in a patient with a history of cardiovascular disease. Preoperative panoramic view (**A**). Immediate postoperative panoramic view after #35i–37i implants installation (**B**). Panoramic view after functional loading (**C**). A 5-year follow-up view after functional loading (**D**). A 10-year follow-up view after functional loading (**E**). In the last visit, the implants were in a good state with very little bone loss observed

**Fig. 7:**
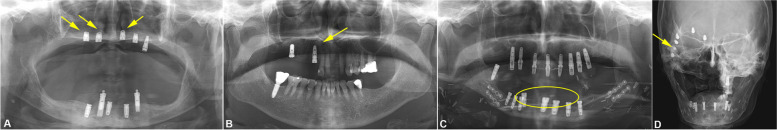
Representative cases among the failed 31 implants out of 1019 total included patients with a history of cardiovascular disease; diabetes mellitus; osteoporosis with #12i, 13i, and 23i failed implants (**A**); hypothyroidism, thalassemia with #13i failed implant (**B**); history of oral and stomach cancer with fibula free flap reconstruction with #42, 41, 31, and 32 failed implants (**C**); and history of spindle cell carcinoma and facial implant failure (**D**) (marked with yellow arrowheads and circle)

In this study, we included patients with general conditions such as CVD including hypertension, DM, and osteoporosis or local conditions such as osteomyelitis; these cases showed good implant initial stability and comparable marginal bone loss compared with implants placed in normal patients (Fig. 6). Representative cases among 31 failed implants out of 1019 included patients with CVD; DM; osteoporosis with #12i, 13i, and 23i failed implants; hypothyroidism; thalassemia with #13i failed implant; history of oral and stomach cancer with fibula free flap reconstruction with #42, 41, 31, and 32 failed implants; history of spindle cell carcinoma; and facial implant failure (Fig. 7).

## Conclusions

Success and survival rates of dental implants in medically compromised patients were comparable with healthy patients. Preoperative general health assessments including laboratory test results and checking of the previous medication records are essential in diagnosing any unrecognized conditions for improved implant success and rates. Regular follow-up is crucial for medically compromised patients, particularly those with a history of conditions such as oral cancer, DM, CVD, hyperlipidemia, or renal disease, as they are more susceptible to experiencing peri-implant health issues due to reduced salivary flow.

## Data Availability

This study was conducted as a retrospective research method, and raw data can be provided at the request of the journal.

## Abbreviations

ASA: American Society of Anesthesiologists
CVD: Cardiovascular disease
DM: Diabetes mellitus
ICOI: International Congress of Oral Implantologists
ITI: International Team for Implantology
SE: Secondary electron
SEM: Scanning electron microscopy
